# Acupuncture as adjunctive therapy for acute cerebral infarction: a randomized clinical trial

**DOI:** 10.3389/fneur.2025.1509204

**Published:** 2025-04-11

**Authors:** Jiang-Peng Cao, Xin-Yue Du, Xiao-Xi Liu, Meng-Han Li, Man Zhang, Sheng-Xuan Guo, Qiu-Han Cai, Jia-Xin Zhang, Shan-Shan Sun, Jia-Wei Han, Lin-Ling Chen, Na Zheng, Lan-Yu Jia, Gui-Ping Li, Yuan-Hao Du

**Affiliations:** ^1^Department of Acupuncture and Moxibustion, First Teaching Hospital of Tianjin University of Traditional Chinese Medicine, Tianjin, China; ^2^National Clinical Research Center for Chinese Medicine Acupuncture and Moxibustion, Tianjin, China; ^3^Department of Graduate School, Tianjin University of Traditional Chinese Medicine, Tianjin, China; ^4^Department of Graduate School, Heilongjiang University of Chinese Medicine, Harbin, China; ^5^The Second Affiliated Hospital of Zhejiang University of Traditional Chinese Medicine, Hangzhou, China; ^6^First Hospital of Jilin University, Changchun, China; ^7^Huzhou Central Hospital, Huzhou, China; ^8^Tianjin Huanhu Hospital, Tianjin, China; ^9^Tianjin Academy of Traditional Chinese Medicine Affiliated Hospital, Tianjin, China

**Keywords:** acute cerebral infarction, acupuncture, efficacy, safety, randomized controlled trial

## Abstract

**Background:**

Acute cerebral infarction (ACI) is the second leading cause of death and the major cause of disability worldwide, and there is an increasing interest in non-pharmacological treatments. Acupuncture has promising effects on ACI, but its efficacy and safety need to be verified through well-designed randomized clinical trials. We aimed to investigate the efficacy and safety of acupuncture as adjunctive therapy to improve neurological function in patients with ACI.

**Methods:**

The multicenter, sham-controlled, patient- and assessor-blinded randomized controlled trial was conducted in 4 tertiary hospitals in China from January to September 2024. All participants received standard care as recommended by the guidelines and were randomly assigned (1:1:1) to manual acupuncture (MA), sham acupuncture (SA), or standard care (SC) only. Participants in the MA and SA groups received acupuncture treatment 6 times weekly for 2 weeks for a total of 12 sessions. The primary outcome was the change in the National Institutes of Health Stroke Scale score from baseline to 14 days. Safety outcomes included adverse events and serious adverse events.

**Results:**

A total of 132 patients (median [IQR] age, 65 [58–69] years; 96 men [72.73%]), with a median (IQR) baseline National Institutes of Health Stroke Scale score of 11 (9–12) points, were included in the intention-to-treat analysis. Ten patients withdrew during the 14-day intervention, and another 7 patients withdrew during the 90-day follow-up. During the 14-day intervention, the median neurological impairment was significantly improved in the MA group compared to the SA group (4 [3, 5] vs. 3 [1.25, 4] points; Cohen’s *d*, 0.76; 95% CI, 0.33 to 1.19; *p* = 0.001). Adverse events occurred relatively equally between the MA and SA groups (19 [43.2%] vs. 13 [29.5%]; relative risk, 1.46; 95% CI, 0.83 to 2.58; *p* = 0.184).

**Conclusion:**

Twelve sessions of MA were safe and effective in improving the neurological function of patients with ACI. The results of this trial indicate that MA can be recommended as a routine, supplemental therapy for improving neurological function in patients with ACI.

**Clinical trial registration:**

ChiCTR2300079204 (Chinese Clinical Trial Registry, http://www.chictr.org.cn, registered on 27/12/2023).

## 1 Introduction

Given its high morbidity, disability, and mortality, stroke remains the second leading cause of death and disability worldwide, significantly contributing to the global burden of diseases (1). As to ischemic stroke, which accounts for 87% of all strokes (2), current evidence-based treatments, such as recombinant tissue plasminogen activator and endovascular thrombectomy, have demonstrated substantial progress over the past few decades (3–5). However, they have several drawbacks, including but not limited to the narrow therapeutic time window (within 4.5 h in recombinant tissue plasminogen activator and within 6 h in endovascular thrombectomy) (6), the risk of systemic bleeding and intracranial hemorrhage, and the low recanalization rate (<10% within 1 h, <35% within 2 h, and < 43% within 24 h) (7), restricting current treatment to only approximately 5% of patients (8). Therefore, there is a need to investigate effective and safe alternative interventions to restore blood flow to the brain promptly and limit neurological injury.

Acupuncture, a complementary and alternative therapy, has been used for treating stroke for over 1,000 years. Experimental studies have shown that acupuncture could suppress endoplasmic reticulum stress (9), reduce inflammatory reactions (10), mediate blood–brain barrier opening and inhibit excitatory toxicity (11), and promote angiogenesis by acting on the coordinated neuro-glial-vascular protection process (12), thus exerting a neuroprotective effect. However, the potential additional positive effect of acupuncture for ACI patients is controversial. Several clinical studies have indicated the safety and efficacy of acupuncture intervention in treating ACI (13–15), whereas some studies showed limited or no beneficial effect of acupuncture as an adjunct treatment to routine management (16–18). In addition, the positive results have yet to show convincing evidence of benefit because of the small sample size, single-center design, unclear methods of randomization and allocation concealment, and no sham-controlled group setting (19, 20). Therefore, this patient- and assessor-blinded, sham-controlled, multicenter study was conducted to evaluate the efficacy and safety of manual acupuncture (MA) in addition to standard care (SC) in patients with ACI. We hypothesized that MA coupled with SC could improve neurological function in patients with ACI compared to sham acupuncture (SA).

## 2 Methods

### 2.1 Study design

This multicenter, sham-controlled, patient- and assessor-blinded, randomized controlled trial was performed at four tertiary hospitals in China from January to September 2024. The study protocol was approved by the Institutional Review Board of the First Teaching Hospital of Tianjin University of Traditional Chinese Medicine and has been previously published (21). The trial was conducted in accordance with the principles of the Declaration of Helsinki (22) and followed the Consolidated Standards of Reporting Trials (Supplementary material, CONSORT-2010-checklist) reporting guideline (23) as well as the Standards for Reporting Interventions in Controlled Trials of Acupuncture guidelines (24). Written informed consent for participation was provided by the patients or their legal representative before randomization.

### 2.2 Participant recruitment

Patients were eligible for inclusion if they were aged 40 to 75 years, had a clinical diagnosis of ischemic stroke within 3 days of symptom onset, and had a National Institutes of Health Stroke Scale (NIHSS) score ranging from 5 to 15 (range, 0–42, with higher scores indicating greater severity). The main exclusion criteria included the neurological deficits resulting from craniocerebral trauma, tumors, and other etiologies, as well as any contraindications to acupuncture. Details of the inclusion and exclusion criteria are provided in Supplementary Table 1.

### 2.3 Randomization and blinding

Eligible patients were randomized to one of the three trial arms (MA group, SA group, and SC group) according to the ratio of 1:1:1, with a fixed block size of 6. Randomization was stratified by the recruitment site and performed through sequentially numbered, opaque, sealed envelopes with full allocation concealment.

The patients in the acupuncture groups were blinded to which acupuncture method they would receive. Due to the particularity of acupuncture treatment, it is impossible to blind the acupuncturists in this study. The outcome assessors, data collectors, and statisticians were blinded to group allocations during the study.

### 2.4 Interventions

In accordance with the Chinese guidelines for the diagnosis and treatment of acute ischemic stroke 2018 (25), we provided SC protocol to all three groups in terms of antiplatelet aggregation or anticoagulant therapy, statin therapy, and control of risk factors regarding ACI.

**Figure 1:**
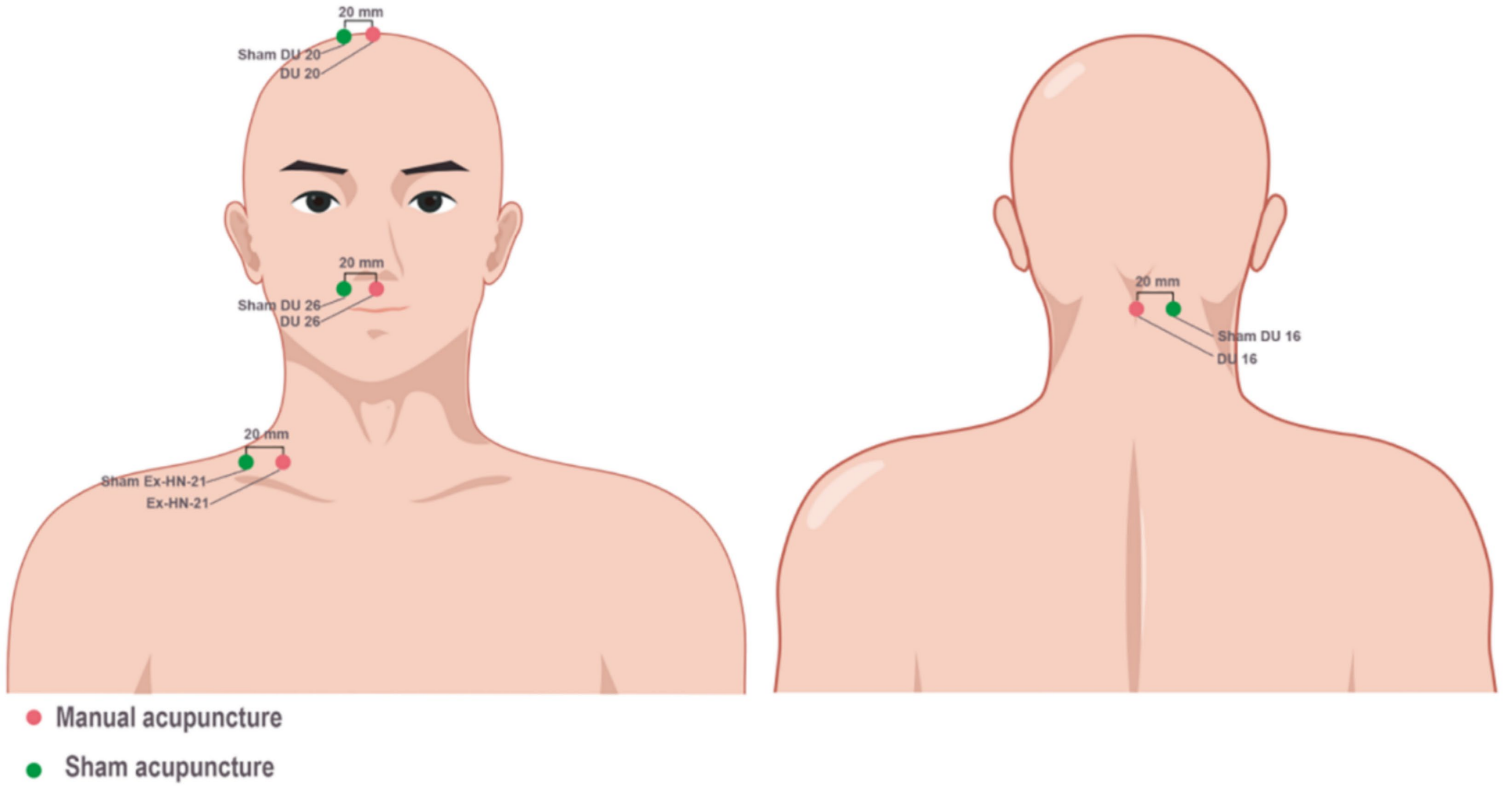
Location of acupoints for the manual acupuncture and sham acupuncture groups.

Acupuncture was performed by eight acupuncturists who had been trained for at least 3 years, have a master’s degree with more than 5 years of clinical experience, and attended centralized training before recruitment. Patients in the MA and SA groups received 12 sessions of acupuncture treatment (6 times weekly for 2 weeks, 30 min per session). Sterile, single-use filiform needles (0.25 mm × 25 mm and 0.25 mm × 40 mm; Hwato brand manufactured by Suzhou Medical Appliance in Jiangsu Province of China) were used in acupuncture treatments. Patients in the MA and SA groups were asked to adopt a lateral position, breathe normally, and relax their whole body for 2 min, and after skin disinfection, they received acupuncture at four fixed acupoints: Renzhong (GV26), Baihui (GV20), Fengfu (GV16), and Jingbi (Ex-HN-21; affected side), or received SA at sham GV26, sham GV20, sham GV16, and sham Ex-HN-21 points (affected side) (Figure 1). Details of acupuncture (location, depth, and manipulation) are shown in Supplementary Table 2.

### 2.5 Outcomes

The primary outcome was the change in the NIHSS score from baseline to Week 2. Secondary outcomes included the Fugl-Meyer Assessment (FMA) scale scores and the Barthel Index (BI). In addition, the modified Rankin Scale (mRS) was also assessed when the follow-up period ended (at 90 days). Safety was assessed, and adverse events that occurred or worsened during treatment or posttreatment were recorded. Serious adverse events were immediately reported to the principal investigator (YH. Du) as well as to the institutional review board within 24 h. All adverse events were followed up until resolution (refer to the procedure diagram in Supplementary Table 3).

### 2.6 Statistical analysis

Based on a previous study (26), a sample size of 36 patients per group was estimated to provide 80% power and a one-sided significance level of 2.5%. An additional 20% was added, considering follow-up losses, and the sample size was increased to 44 patients in each group.

The baseline characteristics and outcomes were analyzed according to the intention-to-treat principle. Missing data were replaced using the last-observation-carried-forward method. Continuous variables were presented as mean (SD) or median (IQR) according to the distribution, and categorical variables were presented as frequency and proportion. The Shapiro–Wilk test and box plots were used to assess the homogeneity of the quantitative variables. For tests across groups, we used the Kruskal–Wallis test when relevant. Normally distributed continuous data were analyzed using a two-tailed t-test, while skewed data were analyzed using the Mann–Whitney U-test. Pearson’s Chi-squared test or Fisher’s exact test was employed to analyze dichotomous data. A two-sided *P* < 0.05 was deemed to be statistically significant. Effect sizes for continuous variables were determined using the standardized mean difference (Cohen’s d), and for dichotomous variables, the effect size was calculated as relative risk. If the global test among the 3 groups was significant, the Bonferroni-adjusted was used for post hoc analysis owing to the data distributions. Analyses were performed with the SPSS statistical software version 27.0 (IBM, Inc.) and Stata statistical software version 12.0 (Stata Corp).

## 3 Results

### 3.1 Patients and characteristics

**Figure 2:**
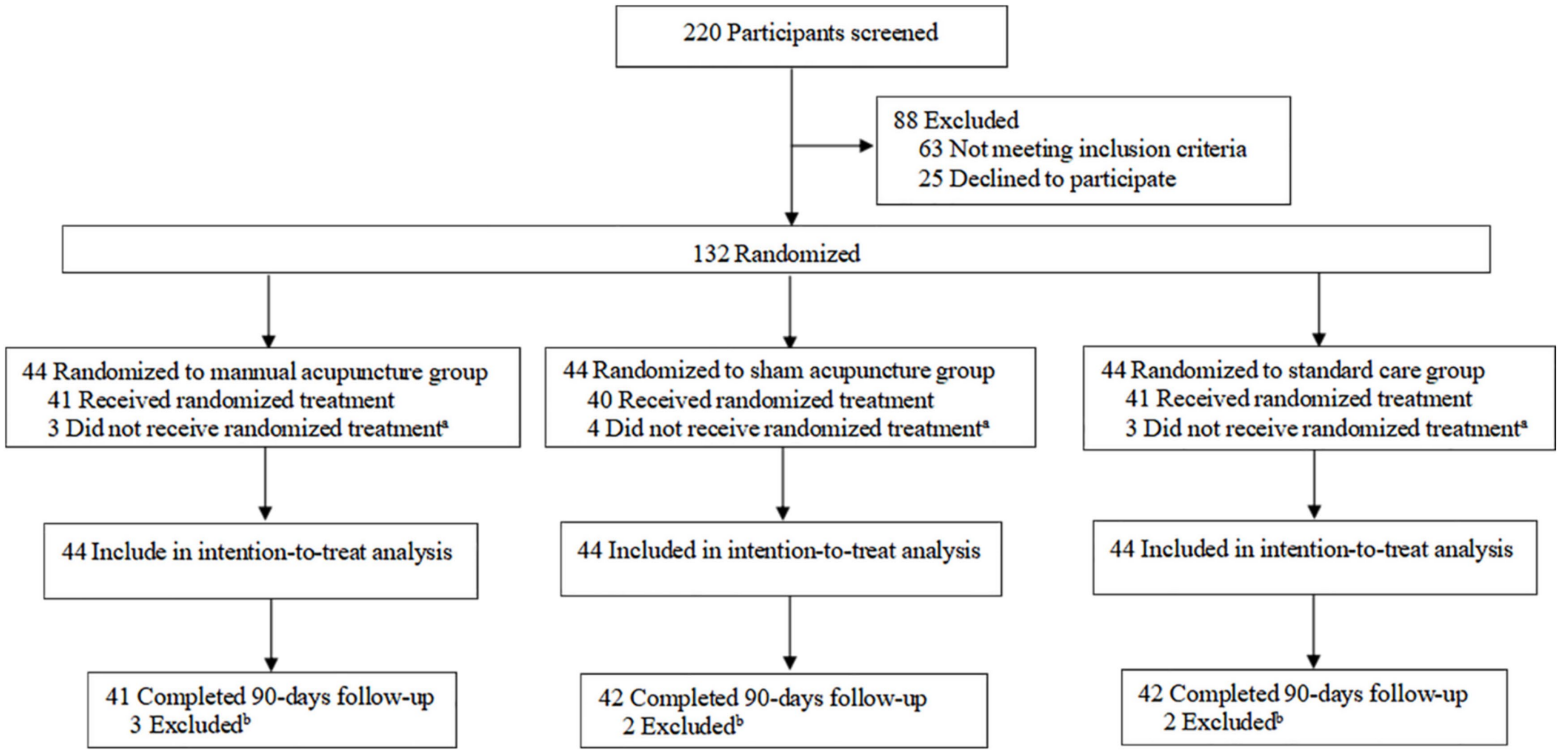
Study flow diagram. ^a^A total of 10 participants did not receive the randomized treatment: two withdrew due to adverse events and one was lost to follow-up in the manual acupuncture group; two withdrew due to adverse events and two were lost to follow-up in the sham acupuncture group; and two withdrew due to adverse events and one was lost to follow-up in the standard care group. ^b^A total of seven participants were excluded: three were lost to follow-up in the manual acupuncture group; two were lost to follow-up in the sham acupuncture group; and two were lost to follow-up in the standard care group.

From January to September 2024, we screened 220 patients, and 88 were excluded due to ineligibility or unwillingness to participate. A total of 44 patients were assigned to receive MA, 44 were assigned to receive SA, and 44 were assigned to receive SC. Among the randomized participants, 115 (87.1%) completed the study. The records of 132 patients were included in the intention-to-treat analysis, and 17 patients (12.8%) dropped out (MA group, 6 [13.6%]; SA group, 6 [13.6%]; SC group, 5 [11.4%]) (Figure 2; Supplementary Table 4).

The demographic and clinical characteristics were comparable among the three groups (Table 1). The median (IQR) age of the patients was 65 (58–69) years, and 96 (72.73%) of the participants were male. The median (IQR) baseline NIHSS score was 11 (9–12) points across all patients. The median (IQR) time from stroke onset to treatment was 2 (1–2) days.

**Table 1:** Baseline characteristics of participants.

Characteristic	Treatment group			*p*-value
	MA (*n* = 44)	SA (*n* = 44)	SC (*n* = 44)	
Age, median (IQR), y	65.00 [57.00, 71.50]	65.00 [59.00, 68.75]	64.00 [56.00, 69.00]	0.888
Sex				
Female	10 (22.73%)	12 (27.27%)	14 (31.82%)	0.632
Male	34 (77.27%)	32 (72.73%)	30 (68.18%)	
BMI, median (IQR)^a^	25.08 [23.05, 27.55]	25.98 [24.20, 28.38]	25.65 [23.96, 27.68]	0.306
Marital status				
Single	5 (11.36%)	4 (9.09%)	8 (18.18%)	0.416
Not single	39 (88.64%)	40 (90.91%)	36 (81.82%)	
Medical history				
Current smoker	29 (65.91%)	21 (47.73%)	18 (40.91%)	0.053
Current drinker	19 (43.18%)	22 (50.00%)	13 (29.55%)	0.139
Previous stroke	16 (36.36%)	12 (27.27%)	10 (22.73%)	0.355
Hypertension	30 (68.18%)	31 (70.45%)	30 (68.18%)	0.965
Diabetes	17 (38.64%)	19 (43.18%)	19 (43.18%)	0.883
Heart disease^b^	13 (29.55%)	10 (22.73%)	9 (20.45%)	0.585
Course of disease, median (IQR), d	2.00 [1.00, 2.00]	2.00 [1.00, 2.00]	2.00 [1.00, 2.00]	0.367
Assessments				
NIHSS score, median (IQR)^c^	10.50 [8.00, 12.00]	11.00 [9.00, 13.00]	10.00 [8.25, 12.00]	0.460
FMA score, mean (SD)^d^	49.59 (8.35)	51.59 (9.45)	49.89 (6.99)	0.479
FMA-UE score, median (IQR)	31.00 [24.25, 37.75]	32.00 [28.00, 36.00]	31.00 [27.00, 33.00]	0.452
FMA-LE score, median (IQR)	18.00 [15.00, 22.00]	18.00 [14.25, 24.75]	18.00 [15.00, 22.75]	0.843
BI, median (IQR)^e^	50.00 [36.25, 50.00]	50.00 [40.00, 55.00]	45.00 [40.00, 53.75]	0.336
mRS, median (IQR)^f^	3.00 [2.75, 4.00]	3.00 [2.00, 4.00]	3.00 [3.00, 4.00]	0.828

BI, Barthel Index; BMI, body mass index; FMA, Fugl-Meyer Assessment; FMA-LE, FMA scale score-lower extremity; FMA-UE, FMA scale-upper extremity; MA, manual acupuncture; mRS, Modified Rankin Scale; NIHSS, National Institute of Health Stroke Scale; SA, sham acupuncture; SC, standard care. ^a^Body mass index is calculated as weight in kilograms divided by height in meters squared. ^b^Heart disease was defined as a range of major clinical heart and circulatory disease conditions, including but not limited to rhythm disorders, coronary artery disease, heart failure, and valvular disease. ^c^Scores range from 0 to 42, with higher scores indicating more serious neurological impairment. ^d^Scores range from 0 to 100, including upper extremity (FMA-UE, scores range from 0 to 66) and lower extremity (FMA-LE, scores range from 0 to 34), with higher scores indicating better motor performance. ^e^A tool to assess functional independence to perform 10 daily activities, scores range from 0 to 100. ^f^A global stroke disability scale with scores ranging from 0 (no symptoms or completely recovered) to 6 (death).

### 3.2 Primary outcome

At the end of the 12-session intervention, the median (IQR) NIHSS score was significantly improved in the MA group compared to the SA group (4 [3, 5] vs. 3 [1.25, 4] points; Cohen’s *d*, 0.76; 95% CI, 0.33–1.19; *p* = 0.001), suggesting that MA was associated with greater improvements in neurologic deficits (Table 2). There was no significant difference between the SA group and the SC group (*p* = 0.144). Similar results were observed in the per-protocol cohort, with 38 patients (86.4%) in the MA group and 38 (86.4%) in the SA group (5 [3.75, 5.25] vs. 3 [2, 4.25] points; Cohen’s *d*, 0.83; 95% CI, 0.36–1.29; *p* = 0.001) (Supplementary Table 5).

**Table 2:** Primary and secondary outcomes in the intention-to-treat cohort.

Outcomes	Intervention			*p*-value*^a^*	Pairwise comparisons			
					MA vs. SA		SA vs. SC	
	MA (*n* = 44)	SA (*n* = 44)	SC (*n* = 44)		Effect size (95% CI)	*p*-value^b^	Effect size (95% CI)	*p*-value^b^
Primary outcome								
NIHSS score, median (IQR)								
Baseline	10.5 [8, 12]	11 [9, 13]	10 [8.25, 12]	0.460	NA	NA	NA	NA
Day 14	6 [4.25, 8]	8 [7, 9]	8 [7, 10]	< 0.001	−1.04 [−1.48 to −0.59]	< 0.001	−0.08 [−0.50 to 0.34]	0.940
Change	4 [3, 5]	3 [1.25, 4]	2 [1, 4]	< 0.001	0.76 [0.33 to 1.19]	0.001	0.29 [−0.13 to 0.71]	0.144
Secondary outcomes								
FMA score, median (IQR)								
Baseline	49.59 (8.35)	51.59 (9.45)	49.89 (6.99)	0.479	NA	NA	NA	NA
Day 14	66 [61.25, 71]	58.5 [51.25, 65.75]	57 [51.25, 61.75]	< 0.001	0.88 [0.44 to 1.32]	< 0.001	0.23 [−0.19 to 0.64]	0.259
Change	−16 [−19, −13]	−7 [−8, −5]	−6.5 [−8, −5]	< 0.001	−2.6 [−3.2 to −2.03]	< 0.001	0.27 [−0.16 to 0.69]	0.966
BI, median (IQR)								
Baseline	50 [36.25, 50]	50 [40, 55]	45 [40, 53.75]	0.336	NA	NA	NA	NA
Day 14	75 [70, 80]	70 [65, 75]	70 [65, 70]	< 0.001	0.51 [0.09 to 0.94]	0.010	0.30 [−0.13 to 0.71]	0.150
Change	−27.5 [−35, −21.25]	−25 [−28.75, −15]	−25 [−30, −20]	0.003	−0.76 [−1.19 to −0.32]	0.001	0.14 [−0.28 to 0.56]	0.553
mRS score, median (IQR)								
Baseline	3 [2.25, 4]	3 [2, 4]	3 [3, 4]	0.828	NA	NA	NA	NA
Day 90	2 [1, 2]	2 [1, 3]	2 [1.25, 3]	0.145	−0.28 [−0.70 to 0.14]	0.204	−0.15 [−0.57 to 0.27]	0.530
Change	2 [1, 2]	1 [1, 1]	1 [1, 1]	< 0.001	0.96 [0.52 to 1.40]	< 0.001	0.31 [−0.11 to 0.73]	0.355

^ap-values based on Kruskal–Wallis analysis among the three groups. bp-values based on Mann–Whitney U-test, and a two-sided p < 0.016 was deemed to be statistically significant applying the Bonferroni adjustment. BI, Barthel Index; FMA, Fugl-Meyer Assessment; MA, manual acupuncture; mRS, Modified Rankin Scale; NIHSS, National Institute of Health Stroke Scale; SA, sham acupuncture; SC, standard care.^

### 3.3 Secondary outcomes

Regarding the secondary efficacy outcome, a significant change in the median FMA score of the MA group was observed at 14 days, compared to the SA group (−16 [−19, −13] vs. -7 [−8, −5] points; Cohen’s *d*, −2.6; 95% CI, −3.2 to −2.03; *p* < 0.001); and there was no significant difference between the SA group and the SC group (*p* = 0.966) (Table 2). Additionally, the MA group performed significantly better on the median FMA-UE score compared to the SA group (−10.5 [−14, −8] vs. -4 [−5, −3] points; Cohen’s *d*, −1.95; 95% CI, −2.45 to −1.43; *p* < 0.001); and there was no significant difference between the SA group and the SC group (*p* = 0.322). Of note, although there was no difference in the median FMA-LE score between the groups at 14 days, an improvement in the median FMA-LE score of the MA group was observed in the difference from baseline to 14 days, compared to the SA group (−5 [−6, −4] vs. −3 [−3, −2] points; Cohen’s *d*, −2.58; 95% CI, −3.15 to −2.01; *p* < 0.001) and there was no significant difference between the SA group and the SC group (*p* = 0.551) (Supplementary Table 6).

At the end of the 14-day intervention, the MA group performed significantly better on the median BI compared to the SA group (−27.5 [−35, −21.25] vs. −25 [−28.75, −15] points; Cohen’s *d*, −0.76; 95% CI, −1.19 to −0.32; *p* = 0.001); and there was no significant difference between the SA group and the SC group (*p* = 0.533) (Table 2).

**Figure 3:**
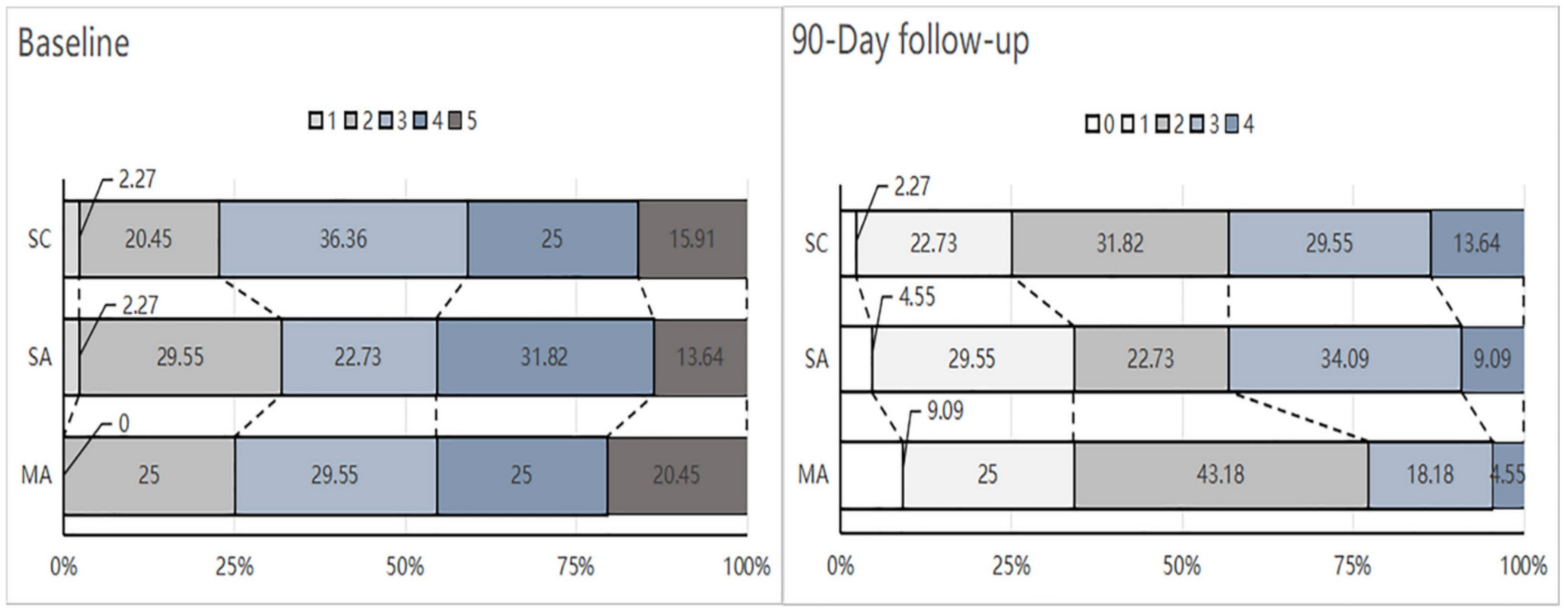
Distribution modified Rankin Scale (mRS) scores at baseline and 90-Day. MA, manual acupuncture; SA, sham acupuncture; SC, standard care. The mRS is a global stroke disability scale with scores ranging from 0 (no symptoms or completely recovered) to 6 (death). None of the participants died during the study period. The numbers in the bars indicate the percentage of patients with each score at baseline and 90-day follow-up for those randomized to the MA (*n* = 44), SA (*n* = 44). or SC (*n* = 44) groups.

Table 2 and Figure 3 show the baseline and 90-day posttreatment results for the mRS score. There was no significant difference in the mRS score at 90 days between the MA group and the SA group (*p* = 0.204), or between the SA group and the SC group (*p* = 0.530). However, it is noteworthy that the difference in improvement from baseline to 90 days was significant between the MA and SA groups (2 [1, 2] vs. 1 [1, 1] points; Cohen’s *d*, 0.96; 95% CI, 0.52 to 1.40; *p* < 0.001), and no significant difference between the SA and SC groups (*p* = 0.355). The proportion of patients with a favorable functional outcome (mRS score of 0–2) in the MA group was 77.3% compared to 56.8% in the SA group (RR, 1.36; 95% CI, 1.00 to 1.84; *p* = 0.041). There were no significant differences between the SA and SC groups (56.8% vs. 56.8; RR, 1; 95% CI, 0.70 to 1.44; *p* = 1). The groups showed no significant difference in the proportion of an mRS score of 0 to 1 at 90 days between the MA and SA groups (*p* = 1) or between the SA and SC groups (*p* = 0.350) (Supplementary Tables 7, 8).

Similar results regarding the secondary efficacy outcomes were observed in the per-protocol cohort (Supplementary Tables 9–12; Supplementary Figure 1).

### 3.4 Adverse events

There was no significant difference in adverse events directly related to acupuncture between the MA group and the SA group (16 [36.36%] vs. 8 [18.18%]; RR, 2; 95% CI, 0.955 to 4.186; *p* = 0.056). The common adverse events directly related to acupuncture are dizziness, pain (moderate or severe), bleeding at acupoints, and local hematoma. There is a similar incidence of severe adverse events between the MA group and the SA group (3 [6.82%] vs. 5 [11.36%]; RR, 0.6; 95% CI, 0.153 to 2.359; *p* = 0.713), as well as between the SA group and the SC group (5 [11.36%] vs. 7 [15.91%]; RR, 0.714; 95% CI, 0.245 to 2.080; *p* = 0.534) (Supplementary Table 13).

### 3.5 Quality of blinding

Sixteen patients (39.02%) in the MA group vs. 14 (35.00%) in the SA group believed that they received verum acupuncture, whereas 19 patients (46.34%) in the MA group and 17 patients (42.50%) in the SA group could not identify their group assignment. No difference was found between the groups in the proportion of patients who correctly guessed the type of acupuncture they had received (*p* = 0.098), suggesting that the blinding was successful (Supplementary Table 14).

## 4 Discussion

In this study, we report results from the randomized controlled trial that assessed MA as an add-on therapy in ACI, coupled with SC, within 3 days from symptom onset. The results showed that MA, as an add-on therapy to SC, was associated with improved recovery of neurological function compared to SA. The results of the secondary outcomes analysis were also consistent and supported the effectiveness of MA. Additionally, both MA and SA groups had similar safety outcomes, including severe adverse events. In sum, our findings provide supportive clinical evidence of the application of MA in the treatment of patients with ACI.

There are a few interventions for ACI at present, with intravenous alteplase being the only one approved by the U.S. Food and Drug Administration in 1996, and it has since become the first-line treatment (27). Antiplatelet therapies have become the most common treatment for patients with ACI in China, partly due to the drawbacks of reperfusion therapy (28, 29). In addition, despite clinical trials that have not yet shown convincing evidence of the benefits of ACI, it is widely used in clinical practice in China. The primary aim for the treatment of ACI is to restore blood supply to salvageable brain tissue in a timely manner, which is also the focus of our team’s long-standing research. Our previous study found that angiogenesis occurs at the border of the ipsilateral ischemic hemisphere after middle cerebral artery occlusion (MCAO), and this self-restoration phenomenon is enhanced by acupuncture stimulation, as indicated by increased endothelial cell proliferation (12). We further explored the related mechanisms, and the results indicated that acupuncture may activate the Ang/Tie system, promoting early blood vessel reconstruction in the ischemic penumbra (30). Additionally, we identified a severe movement disorder in microvascular vasomotor function within the MCAO model, characterized by a “high-speed and low-efficiency shock” phenomenon, which hinders the acquisition of compensatory blood flow to the ischemic region. We have termed this the “microvascular pivot theory,” positing that the functional status of micro-vessels in the cerebral ischemic area is crucial, acting as a “gate” for obtaining peripheral collateral compensatory blood flow. This discovery is closely associated with the cerebral no-reflow phenomenon, which describes the incomplete restoration of downstream microcirculation following recanalization treatment (31, 32), a concept validated by over half a century of scientific research (33–35). Notably, our findings indicate that acupuncture intervention exerts a positive regulatory effect on cerebral vascular smooth muscle cells function through the phosphatidylinositol system and myosin light chain kinase pathway (36–38). A meta-analysis suggested that the quality of evidence in the outcomes of NIHSS, BI, and the FMA total was high; however, there was a risk of bias (lack of concealment and blinding) in these studies, which should be considered when rating down the quality of evidence further (39). Based on the above background, we conducted this multicenter, sham-controlled, patient- and assessor-blinded, randomized controlled trial to evaluate the efficacy of acupuncture in addition to standard care in patients with ACI.

In this randomized clinical trial, we aimed to compare the differences in outcomes from baseline to 14 days between the MA and SA groups. Moreover, there was no difference between the SA and SC groups, indicating the no-efficacy of SA intervention. The primary outcome was the difference in NIHSS scores from baseline to 14 days between the MA and SA groups. The NIHSS was chosen as the primary outcome because it was mainly developed to predict the likelihood of a patient’s recovery of neurological function after a stroke at an early stage. Our findings revealed that the reduction in the NIHSS scores and the increase in BI and FMA scores were greater in the MA group than in the SA group; these results are consistent with several previous trials (40, 41). Although an observer-blinded randomized controlled pilot study did not find significant changes in the NIHSS score between the MA and control groups, it showed a significant reduction within the MA group. Furthermore, the reduction in the NIHSS score in the MA group tended to be greater than that in the control group. Plausible explanations for this outcome may include the small sample size and the stroke severity of patients (mean baseline NIHSS of approximately 4, representing a relatively mild neurological deficit) (42). The effect of acupuncture on mRS was also observed, as a 90-day mRS score of 0–2 indicates a good clinical outcome (43). Although there was no difference at 90 days between the MA and SA groups, the change from the baseline to 90 days was found to be statistically significant. This result differed from a recently published clinical trial (13), partly due to the variety of the acupuncture intervention sessions (8 sessions vs. 12 sessions), sample size (35 cases vs. 88 cases), and the time point of assessment (56 days vs. 90 days). Meanwhile, the safety outcomes did not show a statistical difference between the MA and SA groups. The above results demonstrated that acupuncture intervention was beneficial and safe for the recovery of neurological and motor function in patients with ACI.

To improve the success of blinding the patients, we chose 1 cun (≈20 mm) lateral to the real acupoints as the sham acupoints and kept other factors (number of needles, duration of treatment, and depth) identical to the MA group. The blinding assessment results showed no difference between the MA and SA groups in the proportion of patients who correctly guessed the type of acupuncture they had received (*p* = 0.098) and indicated a low dropout rate, suggesting that blinding was successful in this trial.

Choosing the most appropriate acupoints for stimulation is considered the most important factor in determining the efficacy of acupuncture in clinical practice. Based on the theory of traditional Chinese medicine, we selected three acupoints located in the brain (GV16, GV20, and GV26) from the *Dumai* (concerning its pathway and indications) to awaken the *Brain*, calm the *Shen,* and alleviate *Wind*-induced blockages, along with Ex-HN-21 to improve the motor function of ACI patients. According to the Chinese Yellow Emperor’s Classic of Internal Medicine, an ancient book dating back to 2,600 BC, the *Brain* is considered the Sea of *Marrow*, from the point of GV20 on the head and down to the point of GV16 after the neck. GV26 was first recorded by Ge Hong in A Handbook of Prescriptions for Emergencies, written during the Eastern Jin dynasty (A.D. 283–343), and was widely applied to improve the recovery of consciousness after coma or traumatic brain injuries (44). According to modern physiological and neuroanatomical views, both the GV26 and GV20 acupoints are located within the sensory distribution area (maxillary and ophthalmic) of the trigeminal nerve. There exists a close relationship between the trigeminal nerve and the cerebral vasculature (trigemino-cerebrovascular network), which innervates the majority of the cerebral arteries and significantly contributes to the control of cerebrovascular tone (45). Meanwhile, the GV20 acupoint is also located within the greater occipital nerve, and the GV16 acupoint is distributed with branches of the third occipital nerve and the greater occipital nerve. Several clinical trials demonstrated that the application of acupuncture at GV16, GV20, and GV26 could effectively increase the blood flow volume (46, 47). A few clinical trials have reported that a 10- to 18-session acupuncture treatment benefits neurological function deficits (14, 15, 18). Given the difference in the days after stroke onset and the limited evidence of the specific time frame of rehabilitation for neurological function, we selected a 12-session intervention for the acupuncture treatment. We deduce that the positive results of this trial may be attributed to the neuroanatomical basis behind acupuncture intervention, making it a promising target for the management of ACI. Based on the 12-session intervention over 2 weeks and previous similar study designs, we chose to assess change in the NIHSS score from baseline to Week 2 (12, 42). In addition, the improvement in the 90-day mRS outcomes observed in new treatment studies for ACI is almost universally endorsed by regulatory agencies, such as the U.S. Food and Drug Administration or European clinical guideline committees (6, 48).

The commonly used controls in acupuncture trials include a waitlist or non-intervention control, non-insertion sham control, and needle insertion at sham or real acupoints (49). Patients in this trial all received positive treatment to identify an adjunct effect of acupuncture compared to standard care. We selected the specific sham acupuncture points (1 cun lateral to true points) to control the non-specific effect while keeping other needling components (insertion depth, needle size, needle number, and stimulation) consistent with the MA group, and the results showed that there was no difference between the SA and SC groups in primary and secondary outcomes, which indicated that these points were validated as inactive for the treatment of ACI.

### 4.1 Limitations

First, fewer acupoints were selected for stimulation in the present study to evaluate efficacy and safety, which may cause performance bias. Second, considering the time economic cost and patient adherence (18), we only provided 12 sessions of treatment and assessed outcomes based on relatively short-term measurements. Therefore, future studies need to conduct a longer-term assessment. Third, this trial was conducted in China, mainly involving patients of Han Chinese descent, which may limit the generalizability of the results. Fourth, acupuncturists could not be blinded due to the nature of the interventions, which may introduce performance bias; however, the patients, principal investigator, and statisticians were blinded. They underwent rigorous training on safety and intervention delivery and had consistent fidelity monitoring. Fifth, there was no correction for multiple comparisons for the secondary analyses, and therefore, these findings should be considered exploratory. Sixth, although our results suggest that acupuncture intervention can be initiated within a window of 72 h after the onset of stroke, experimental studies in our laboratory indicate that neurological outcomes are better if acupuncture intervention is initiated within 6 h after the onset of stroke (36). Hence, if acupuncture intervention is initiated quickly after the onset of stroke, a greater improvement in neurological outcome may be possible. Seventh, patients who were included in this trial did not receive intravenous thrombolysis or mechanical thrombectomy. However, given the increasing popularity of recanalization therapy, further research on the efficacy and safety of acupuncture based on successful recanalization is warranted.

## 5 Conclusion

In this randomized clinical trial, MA was associated with improved neurological function in patients with ACI within 72 h of onset, without increasing the risk of safety events. These findings indicate that MA should be considered as an adjunctive treatment option for improving neurological impairment.

## Data Availability

The original contributions presented in the study are included in the article/Supplementary material, further inquiries can be directed to the corresponding author.
