# Supplementary material for: Acupuncture as adjunctive therapy for acute cerebral infarction: a randomized clinical trial
Source: Front Neurol. 2025 Apr 11;16:1509204. doi: 10.3389/fneur.2025.1509204 (PMC12023752; doi:10.3389/fneur.2025.1509204)
Supplement: Supplementary file 1 [file Table_1.docx]

**Supplementary Content**

**Supplementary Table 1.** Inclusion and Exclusion Criteria

**Supplementary Table 2.** Acupuncture Operation Details

**Supplementary Table 3.** The Procedure Diagram

**Supplementary Table 4.** The reasons for patients who did not complete the 90-Day visit

**Supplementary Table 5. Primary outcome in the Per-Protocol Cohort**

**Supplementary Table 6.** Changes in FMA-UE and FMA-LE Score in the Intention-to-Treat Cohort

**Supplementary Table 7.** Distribution mRS Score at Baseline and 90-Day in the Intention-to-Treat Cohort

**Supplementary Table 8.** Proportion of mRS Score at 90-Day in the Intention-to-Treat Cohort

**Supplementary **Table 9.**** Secondary Outcomes in the Per-Protocol Cohort

**Supplementary **Table 10.**** Changes in FMA-UE and FMA-LE Scores in the Per-Protocol Cohort

**Supplementary **Table 11.** Distribution of mRS Score at baseline and 90-Day** in the Per-Protocol Cohort

**Supplementary **Table 12.** Proportion of mRS Score at 90-Day** in the Per-Protocol Cohort

**Supplementary **Fig. 1.** Distribution of mRS Score at Baseline and 90-Day in the Intention-to-Treat Cohort**

**Supplementary **Table 13.**** Adverse Events During Scheduled Acupuncture Treatment Periods

**Supplementary **Table 14.**** Quality of Blinding

This supplementary material has been provided by the authors to give readers additional information about their work.

****Supplementary Table 1. Inclusion and Exclusion Criteria****

| ****Inclusion Criteria**** |
| --- |
| - **Aged 40 to 75 years of age;** - **Within 3 days of stroke onset of ischemic stroke (diagnosis standard by the Chinese guidelines for the diagnosis and treatment of acute ischemic stroke 2018);** - **A National Institutes of Health Stroke Scale score ranging from 5 to 15;** - **Willingness to participate voluntarily and provision of a duly signed informed consent.** |
| ****Exclusion Criteria**** |
| - **Impairment of the Vesta channel’s functionality resulting from craniocerebral trauma, tumors, and other etiologies;** - **Assessment of the impact of acupuncture treatment or other therapeutic medications administered within a 3months period prior to admission;** - **Presence of consciousness disorders, deafness, severe depression, schizophrenia, and inability to cooperate;** - **Coexistence of severe primary diseases affecting the cardiovascular, neurological, respiratory, hepatic, and renal systems;** - **Severe dermatological conditions or hypersensitivity to the investigational treatment;** - **Pregnant, lactating, or expecting women;** - **Patients concurrently participating in other research studies.** |

**Supplementary Table 2. Acupuncture Operation Details**

|  | ****Acupuncture points**** | ****Acupuncture location**** | ****Acupuncture depth**** | ****Acupuncture manipulation**** |
| --- | --- | --- | --- | --- |
| ****Manual acupuncture group**** | **Renzhong (GV26)** | **At the junction of the upper 1/3 and middle 1/3 of the philtrum** | **Insert at a depth of 0.3–0.5 cun**  **(≈6–10 mm)** | **Bird-pecking needling until the eyes become wet or developed tears** |
|  | **Baihui (GV20)** | **At the junction of a line connecting the apices of the ears and the midline, 5 cun [≈100 mm] from the anterior or 7 cun [≈140 mm] from the posterior hairline** | **Insert at an approximately 15⁰ angle to a depth of 0.8–1.2 cun (≈16–24 mm)** | **Rotated for at least 200 revolutions per minute for 1 min** |
|  | **Fengfu (GV16)** | **On the posterior midline, directly below the external occipital protuberance, in the depression between the origins of the trapezius muscle** | **Insert at a depth of 0.8–1.0 cun (≈16–20 mm)** | **Lifting, thrusting, and twirling manipulation to attain a sensation of soreness, aching, heaviness, swelling, or numbness** |
|  | **Jingbi (Ex-HN-21)** | **1 cun [≈20 mm] superior to the junction of the proximal and middle third of the clavicle** | **Insert at a depth of 0.3–0.5 cun (≈6–10 mm)** | **With the lifting manipulation on a small scale and then removed the needle if the sensation obtained (an electric sensation from the arm to the finger).** |
| ****Sham acupuncture group**** | **Sham GV26** | **1 cun (≈20mm) lateral to GV26** | **Insert at a depth of 0.3–0.5 cun**  **(≈6–10 mm)** | **Without needle manipulation** |
|  | **Sham GV20** | **1 cun (≈20mm) lateral to GV20** | **Insert at an approximately 15⁰ angle to a depth of 0.8–1.2 cun (≈16–24 mm)** |  |
|  | **Sham GV16** | **1 cun (≈20mm) lateral to GV16** | **Insert at a depth of 0.8–1.0 cun (≈16–20 mm)** |  |
|  | **Sham Ex-HN-21** | **1 cun (≈20mm) lateral to Ex-HN-21** | **Insert at a depth of 0.3–0.5 cun (≈6–10 mm)** |  |

**Supplementary Table 3. The Procedure Diagram**

|  | | **Screening Phase** | | **Treatment Phase** | | **Follow-up Phase** | |
| --- | --- | --- | --- | --- | --- | --- | --- |
| **Time Points (week)** | **Week -1** | **Baseline** | **After first treatment** | **Week 1** | **Week 2** | **After last treatment** | **Week 12** |
| **Enrollment** | | | | | | | |
| **Eligibility screem** | X |  |  |  |  |  |  |
| **Informed consent** | X |  |  |  |  |  |  |
| **Randomization** |  | X |  |  |  |  |  |
| **Interventions** | | | | | | | |
| **MA** |  |  |  |  |  |  |  |
| **SA** |  |  |  |  |  |  |  |
| **SC** |  |  |  |  |  |  |  |
| **Assessment** | | | | | | | |
| **NIHSS** |  | X |  |  | X |  |  |
| **FMA** |  | X |  |  | X |  |  |
| **BI** |  | X |  |  | X |  |  |
| **mRS** |  | X |  |  | X |  | X |
| **Blinding assessment** |  |  |  |  |  | X |  |
| **Adverse events** |  |  |  |  |  |  |  |

Abbreviations: BI, Barthel Index; FMA, Fugl-Meyer Assessment; MA, manual acupuncture; mRS, Modified Rankin Scale; NIHSS, National Institute of Health Stroke Scale; SA, sham acupuncture; SC, standard care

**Supplementary Table 4. The reasons for patients who did not complete the 90-Day visit**

|  | **Patients, No. (%)** | | | |
| --- | --- | --- | --- | --- |
|  | **MA (n=44)** | **SA (n=44)** | **SC (n=44)** | **Total (n=132)** |
| **Adverse events** | **2 (4.5)** | **2 (4.5)** | **2 (4.5)** | **6 (4.5)** |
| **Loss of follow-up** | **4 (9.1)** | **4 (9.1)** | **3 (6.8)** | **11 (8.3)** |

Abbreviations: MA, manual acupuncture; SA, sham acupuncture; SC, standard care

****Supplementary Table 5. Primary outcome in the Per-Protocol Cohort****

| NIHSS score, median (IQR) | Intervention | | | MA vs SA | | SA vs SC | |
| --- | --- | --- | --- | --- | --- | --- | --- |
|  | MA (n = 38) | SA (n = 38) | SC (n = 37) | Effect size  (95% CI) | *P* value | Effect size  (95% CI) | *P* value |
| Baseline | 11 [9, 12.25] | 11 [9, 13] | 11 [9, 12] | -0.13 [-0.57 to 0.33] | .564 | 0.15 [-0.31 to 0.60] | .432 |
| Day 14 | 6 [5, 8] | 8 [7, 9] | 8 [6.5, 10] | -1.05 [-1.53 to -0.57] | < .001 | -0.12 [-0.58 to 0.33] | .834 |
| Change | 5 [3.75, 5.25] | 3 [2, 4.25] | 2 [1, 4] | 0.83 [0.36 to 1.29] | .001 | 0.25 [-0.21 to 0.70] | .265 |

Abbreviations: MA, manual acupuncture; SA, sham acupuncture; SC, standard care

****Supplementary Table 6. Changes in FMA-UE and FMA-LE Score** in the Intention-to-Treat Cohort**

| Outcomes | Intervention | | | MA vs SA | | SA vs SC | |
| --- | --- | --- | --- | --- | --- | --- | --- |
|  | MA (n = 44) | SA (n = 44) | SC (n = 44) | Effect size  (95% CI) | *P* value | Effect size  (95% CI) | *P* value |
| **FMA-UE score, median (IQR)** | | | | | | | |
| Baseline | 31 [24.25, 37.75] | 32 [28, 36] | 31 [27, 33] | NA | NA | NA | NA |
| Day 14 | 42 [39, 45.75] | 36 [32, 40.75] | 35 [32.25, 37.75] | 0.94 [0.49 to 1.37] | < .001 | 0.33 [-0.10 to 0.75] | .161 |
| Change | -10.5 [-14, -8] | -4 [-5, -3] | -3 [-6, -3] | -1.95 [-2.45 to -1.43] | < .001 | -0.11 [-0.53 to 0.31] | .322 |
| **FMA-LE score, median (IQR)** | | | | | | | |
| Baseline | 18 [15, 22] | 18 [14.25, 24.75] | 18 [15, 22.75] | NA | NA | NA | NA |
| Day 14 | 23 [21, 26.75] | 21 [17, 26.75] | 21 [18, 25] | 0.37 [-0.05 to 0.79] | .083 | 0.01 [-0.40 to 0.43] | .993 |
| Change | -5 [-6, -4] | -3 [-3, -2] | -3 [-3, -2] | -2.58 [-3.15 to -2.01] | < .001 | 0.18 [-0.24 to 0.60] | .551 |

**Abbreviations: FMA, Fugl-Meyer Assessment; FMA-LE, FMA scale score-lower extremity; FMA-UE, FMA scale-upper extremity; MA, manual acupuncture; SA, sham acupuncture; SC, standard care**

****Supplementary Table 7. Distribution mRS Score at Baseline and 90-Day** in the Intention-to-Treat Cohort**

|  | | MA (n = 44) | SA (n = 44) | SC (n = 44) |
| --- | --- | --- | --- | --- |
| Baseline | 1 | 0 (0) | 1 (2.27) | 1 (2.27) |
|  | 2 | 11 (25) | 13 (29.55) | 9 (20.45) |
|  | 3 | 13 (29.55) | 10 (22.73) | 16 (36.36) |
|  | 4 | 11 (25) | 14 (31.82) | 11 (25) |
|  | 5 | 9 (20.45) | 6 (13.64) | 7 (15.91) |
| 90-Day | 0 | 4 (9.09) | 2 (4.55) | 1 (2.27) |
|  | 1 | 11 (25) | 13 (29.55) | 10 (22.73) |
|  | 2 | 19 (43.18) | 10 (22.73) | 14 (31.82) |
|  | 3 | 8 (18.18) | 15 (34.09) | 13 (29.55) |
|  | 4 | 2 (4.55) | 4 (9.09) | 6 (13.64) |

Abbreviations: MA, manual acupuncture; SA, sham acupuncture; SC, standard care

****Supplementary Table 8. Proportion of mRS Score at 90-Day** in the Intention-to-Treat Cohort**

|  | MA  (n = 44) | SA  (n = 44) | SC  (n = 44) | MA vs SA | | SA vs SC | |
| --- | --- | --- | --- | --- | --- | --- | --- |
|  |  |  |  | Effect size  (95% CI) | *P* value | Effect size  (95% CI) | *P* value |
| mRS score of 0 or 1 | 15 (34.1) | 15 (34.1) | 11 (25) | 1 [0.56 to 1.79] | 1 | 1.36 [0.71 to 2.63] | .350 |
| mRS score of ≤ 2 | 34 (77.3) | 25 (56.8) | 25 (56.8) | 1.36 [1.00 to 1.84] | .041 | 1 [0.70 to 1.44] | 1 |

Abbreviations: MA, manual acupuncture; mRS, Modified Rankin Scale; SA, sham acupuncture; SC, standard care

****Supplementary Table 9. Secondary Outcomes in the Per-Protocol Cohort****

| Secondary outcomes | Intervention | | | MA vs SA | | SA vs SC | |
| --- | --- | --- | --- | --- | --- | --- | --- |
|  | MA (n = 38) | SA (n = 38) | SC (n = 37) | Effect size  (95% CI) | *P* value | Effect size  (95% CI) | *P* value |
| **FMA score, median (IQR)** | | | | | | | |
| Baseline | 48.5 [43, 57] | 51.5 [43, 59.25] | 49 [44, 54] | -0.27 [-0.72 to 0.18] | .308 | 0.29 [-0.17 to 0.74] | .332 |
| Day 14 | 66 [61, 71] | 57.5 [51, 67] | 56 [50, 61.5] | 0.81 [0.33 to 1.27] | .002 | 0.31 [-0.15 to 0.76] | .256 |
| Change | -16 [-19, -13] | -7 [-8, -5.75] | -7 [-8.5, -5] | -2.53 [-3.13 to -1.92] | < .001 | -0.01 [-0.46 to 0.44] | .961 |
| **BI, median (IQR)** | | | | | | | |
| Baseline | 47.5 [35, 50] | 50 [40, 55] | 45 [37.5, 52.5] | -0.25 [-0.70 to 0.20] | .298 | 0.34 [-0.12 to 0.80] | .164 |
| Day 14 | 75 [70, 80] | 70 [65, 75] | 70 [65, 70] | 0.56 [0.10 to 1.02] | .013 | 0.24 [-.21 to 0.70] | .343 |
| Change | -30 [-35, -20] | -20 [-26.25, -15] | -25 [-30, -17.5] | -0.87 [-1.34 to -0.40] | < .001 | 0.21 [-0.25 to 0.66] | .400 |
| **mRS score, median (IQR)** | | | | | | | |
| Baseline | 3.5 [2.75, 4.25] | 4 [2, 4] | 3 [2.5, 4] | 0.10 [-0.36 to 0.55] | .727 | 0.04 [-0.41 to 0.49] | .830 |
| Day 90 | 2 [1, 3] | 2.5 [1, 3] | 2 [1, 3] | -0.35 [-0.81 to 0.10] | .133 | -0.03 [-0.49 to 0.42] | .903 |
| Change | 2 [1, 2] | 1 [1, 1] | 1 [1, 1] | 0.99 [0.51 to 1.47] | < .001 | 0.26 [-0.19 to 0.72] | .265 |

**Abbreviations: BI, Barthel Index; FMA, Fugl-Meyer Assessment; MA, manual acupuncture; mRS, Modified Rankin Scale; NIHSS, National Institute of Health Stroke Scale; SA, sham acupuncture; SC, standard care**Supplementary Table 10. Changes in FMA-UE and FMA-LE Scores** in the Per-Protocol Cohort**

| Outcomes | Intervention | | | MA vs SA | | SA vs SC | |
| --- | --- | --- | --- | --- | --- | --- | --- |
|  | MA (n = 38) | SA (n = 38) | SC (n = 37) | Effect size  (95% CI) | *P* value | Effect size  (95% CI) | *P* value |
| **FMA-UE score, median (IQR)** | | | | | | | |
| Baseline | 30 [24, 38] | 32 [27.75, 36] | 30 [26, 32.5] | -0.18 [-0.63 to 0.28] | .396 | 0.34 [-0.12 to 0.80] | .147 |
| Day 14 | 41 [38.75, 46] | 36 [32, 40.25] | 35 [30, 36.5] | 0.93 [0.45 to 1.40] | < .001 | 0.42 [-0.04 to 0.88] | .086 |
| Change | -10.5 [-14, -8] | -4 [-5, -3] | -3 [-6, -3] | -1.88 [-2.41 to -1.33] | < .001 | -0.09 [-0.54 to 0.36] | .417 |
| **FMA-LE score, median (IQR)** | | | | | | | |
| Baseline | 17.5 [14, 22] | 18 [14.75, 25] | 18 [14.5, 23] | -0.24 [-0.69 to 0.21] | .351 | 0.09 [-0.36 to 0.54] | .702 |
| Day 14 | 22.5 [19.75, 28] | 21 [17.75, 27] | 21 [18, 25.5] | 0.26 [-0.19 to 0.71] | .206 | 0.06 [-0.39 to 0.51] | .853 |
| Change | -5 [-6, -4] | -3 [-3, -2] | -3 [-3, -2] | -2.57 [-3.17 to -1.95] | < .001 | 0.19 [-0.27 to 0.64] | .603 |

Abbreviations: FMA, Fugl-Meyer Assessment; FMA-LE, FMA scale score-lower extremity; FMA-UE, FMA scale-upper extremity; MA, manual acupuncture; SA, sham acupuncture; SC, standard care

****Supplementary Table 11. Distribution of mRS Score at baseline and 90-Day** in the Per-Protocol Cohort**

|  | | MA (n = 38) | SA (n = 38) | SC (n = 37) |
| --- | --- | --- | --- | --- |
| Baseline | 1 | 0 (0) | 1 (2.63) | 1 (2.70) |
|  | 2 | 9 (23.68) | 9 (23.68) | 8 (21.62) |
|  | 3 | 10 (26.32) | 8 (21.05) | 11 (29.73) |
|  | 4 | 10 (26.32) | 14 (36.84) | 12 (32.43) |
|  | 5 | 9 (23.68) | 6 (15.79) | 6 (16.22) |
| 90-Day | 0 | 4 (10.53) | 1 (2.63) | 1 (2.70) |
|  | 1 | 7 (18.42) | 10 (26.32) | 9 (24.32) |
|  | 2 | 17 (44.74) | 8 (21.05) | 9 (24.32) |
|  | 3 | 8 (21.05) | 15 (39.47) | 13 (35.14) |
|  | 4 | 2 (5.26) | 4 (10.53) | 5 (13.51) |

**Abbreviations: MA, manual acupuncture; SA, sham acupuncture; SC, standard care**

****Supplementary Table 12. Proportion of mRS Score at 90-Day** in the Per-Protocol Cohort**

|  | MA  (n = 38) | SA  (n = 38) | SC  (n = 37) | MA vs SA | | SA vs SC | |
| --- | --- | --- | --- | --- | --- | --- | --- |
|  |  |  |  | Effect size  (95% CI) | *P* value | Effect size  (95% CI) | *P* value |
| mRS score of 0 or 1 | 11 (28.95) | 11 (28.95) | 10 (27.03) | 1 [0.49 to 2.02] | 1 | 1.07 [0.52 to 2.22] | .853 |
| mRS score of ≤ 2 | 28 (73.68) | 19 (50) | 19 (51.35) | 1.47 [1.02 to 2.13] | .034 | 0.97 [0.62 to 1.52] | .907 |

**Abbreviations: MA, manual acupuncture; mRS, Modified Rankin Scale; SA, sham acupuncture; SC, standard careSupplementary Figure 1. Distribution of mRS Score at Baseline and 90-Day in the Intention-to-Treat Cohort**

| 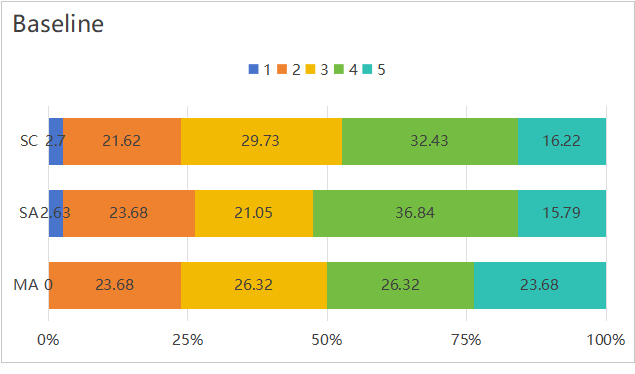 |
| --- |
| 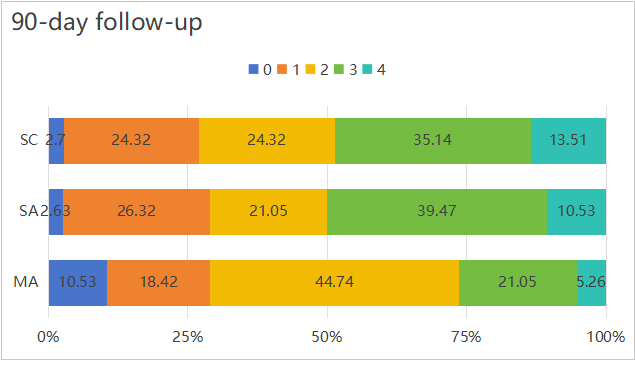 |

**Abbreviations: MA, manual acupuncture; SA, sham acupuncture; SC, standard care**

**The mRS is a global stroke disability scale with scores ranging from 0 (no symptoms or completely recovered) to 6 (death). None of the participants died during the study period. Numbers in the bars indicate the percentage of patients with each score at baseline and 90-Day follow-up for those randomized to MA (n = 38), SA (n = 38) or SC (n = 37). Supplementary Table 13. Adverse Events During Scheduled Acupuncture Treatment Periods**

| Adverse Events | MA  (n = 44) | SA  (n = 44) | SC  (n = 44) | MA vs SA | | SA vs SC | |
| --- | --- | --- | --- | --- | --- | --- | --- |
|  |  |  |  | Effect size (95% CI) | *P* value | Effect size (95% CI) | *P* value |
| Total | 19 (43.2) | 13 (29.5) | - | 1.46 [0.83 to 2.58] | .184 | *-* | |
| Adverse event directly related to acupuncture | | | | | | | |
| Dizziness | 2 (4.5) | 0 (0) | - | 2 [0.96 to 4.19] | .056 | - | - |
| Pain (moderate or severe) | 4 (9.1) | 2 (4.5) | - |  |  |  |  |
| Bleeding at acupoints | 8 (18.2) | 5 (11.4) | - |  |  |  |  |
| Local hematoma | 2 (4.5) | 1 (2.3) | - |  |  |  |  |
| Severe adverse event | | | | | | | |
| Death | 0 (0) | 0 (0) | 0 (0) | 0.60 [0.15 to 2.36] | .713 ^a^ | 0.71 [0.25 to 2.08] | .534 |
| Pneumonia | 1 (2.3) | 1 (2.3) | 2 (4.5) |  |  |  |  |
| Urinary tract infection | 1 (2.3) | 1 (2.3) | 2 (4.5) |  |  |  |  |
| Recurrent stroke | 1 (2.3) | 2 (4.5) | 1 (2.3) |  |  |  |  |
| Alimentary tract hemorrhage | 0 (0) | 1 (2.3) | 1 (2.3) |  |  |  |  |
| Myocardial infarction | 0 (0) | 0 (0) | 1 (2.3) |  |  |  |  |
| Heart failure | 0 (0) | 0 (0) | 0 (0) |  |  |  |  |

Abbreviations: MA, manual acupuncture; SA, sham acupuncture; SC, standard care

^a^ Statistical significance was calculated with Fisher’s exact test.

**Supplementary Table 14. Quality of Blinding**

| Perception of group allocation | No. (%) | | *P* value |
| --- | --- | --- | --- |
|  | MA | SA |  |
| Total patients | 41 | 40 | *-* |
| Real acupuncture | 16 (39.02) | 14 (35.00) | .098 |
| Sham acupuncture | 6 (14.63) | 9 (22.50) |  |
| Do not know | 19 (46.34) | 17 (42.50) |  |

Abbreviations: MA, manual acupuncture; SA, sham acupuncture
